# Puerarin blocks the aging phenotype in human dermal fibroblasts

**DOI:** 10.1371/journal.pone.0249367

**Published:** 2021-04-22

**Authors:** Yuki Kamiya, Mao Odama, Aki Mizuguti, Shigeru Murakami, Takashi Ito

**Affiliations:** Department of Biosciences and Biotechnology, Fukui Prefectural University, Fukui, Japan; University of Sharjah College of Health Sciences, UNITED ARAB EMIRATES

## Abstract

Dermal fibroblast aging contributes to aging-associated functional defects in the skin since dermal fibroblasts maintain skin homeostasis by interacting with the epidermis and extracellular matrix. Here, we found that puerarin, an isoflavone present in *Pueraria lobata* (Kudzu), can prevent the development of the aging-phenotype in human dermal fibroblasts. Normal human dermal fibroblasts (NHDFs) were subcultivated and high-passage cells were selected as senescent cells, whereas low-passage cells were selected as a young cell control. Puerarin treatment increased cell proliferation and decreased the proportion of senescence-associated beta-galactosidase-positive cells in a high-passage culture of NHDFs. Moreover, puerarin treatment reduced the number of smooth muscle actin (SMA)-positive myofibroblasts and the expression of a reticular fibroblast marker, calponin 1 (CNN1), which were induced in high-passage NHDFs. Fulvestrant, an estrogen receptor antagonist, blocked the puerarin-mediated downregulation of SMA and CNN1. Our results suggest that puerarin may be a useful functional food that alleviates aging-related functional defects in dermal fibroblasts.

## Introduction

Skin aging is affected by diet and nutrition as well as other intrinsic factors such as chronological aging, and extrinsic factors such as sun exposure [1, 2]. Since excessive amounts of reactive oxygen species (ROS) generated by harmful stresses cause cellular senescence and aging phenotype, anti-oxidant-rich foods have been investigated as a preventive strategy to delay skin aging [2–4]. Additionally, recent evidence suggests that the targeting of aging-associated changes by natural products can prevent age-related phenotypes [5, 6].

Cellular senescence is a hallmark of normal aging, which is a state of irreversible growth arrest triggered by both endogenous and exogenous stresses [6–8]. Senescent cells are characterized by the induction of senescence-associated beta-galactosidase activity, telomere shortening, and the expression of cyclin-kinase inhibitors such as p16 or p21. Senescent cells secrete proinflammatory cytokines, growth factors, and matrix metalloproteases, that contribute to local and systemic dysfunction [9, 10]. Increasing evidence has demonstrated that the clearance of senescent cells prevents the age-associated decline of tissue function and extends lifespan [11]. Recent investigations have revealed that certain natural compounds called senolytics, can selectively eliminate senescent cells, and these compounds are expected to prevent the age-associated decline of tissue function [6, 12, 13].

Fibroblasts play an important role in maintaining tissue structure via the synthesis of extracellular matrix proteins such as collagen and elastin in most tissues. The skin dermis has three layers: the papillary dermis, the reticular dermis, and the hypodermis [14]. The papillary dermis is a superficial layer that is in contact with the epidermis and has a relatively higher cell density, whereas the reticular dermis is a deeper layer, and has a relatively thicker extracellular matrix and a lower cell density [15, 16]. The fibroblasts residing in the papillary and reticular dermises have distinct identities. Papillary fibroblasts exhibit a spindle-shaped morphology and show higher proliferative activity than that of reticular fibroblasts. Whereas, reticular fibroblasts exhibit a polygonal morphology, and express the myofibroblast marker alpha-smooth muscle actin (SMA) [17]. Aging processes mainly affect the cellular function of papillary fibroblasts, including a decrease in proliferative capacity and an increase in transdifferentiation to form reticular fibroblasts, which may contribute to the aging-associated structural changes [18–21]. Recent studies showed that diet and nutrition can influence the skin phenotype by controlling the aging of dermal fibroblasts [5, 22, 23]; therefore, nutritional agents may be able modify fibroblast function and prevent skin aging.

Puerarin (daidzein-8-C-glucoside) is a predominant isoflavone found in the edible legume *Pueraria lobata* (Kudzu, Kuzu, Gegen) [5, 22–24]. Puerarin is abundant in the vines and roots of *P*. *lobata*. Approximately 0.1–1 g of puerarin is present in 100 g of vines, and 3–10 g is present in 100 g of roots [25–28]. The roots of *P*. *lobata* are also used as a component in traditional Chinese medicine to treat chills, colds, fever, and diarrhea. Recent studies have revealed a variety of pharmacological actions of puerarin, such as vasodilatation, cardioprotection, anticancer, and attenuation of menopausal symptoms [29, 30]. Orally ingested puerarin is absorbed from the gut into the blood; the oral bioavailability of puerarin has been reported to be approximately 4–7% in animal models [31–34]. Regarding the effects on aging, puerarin reduces the endothelial progenitor cell senescence [35]. However, the effects of puerarin on skin aging and fibroblast phenotype remain less understood.

In the present study, we investigated the cytoprotective and anti-aging properties of puerarin in normal human dermal fibroblasts (NHDFs) in vitro.

## Methods

### Cell culture

NHDFs (Catalog number: C-12302, lot number 435Z020.3; PromCell, Heidelberg, Germany) were maintained in Dulbecco’s modified Eagles medium (Nacalai-tesque, Kyoto, Japan) containing 10% fetal bovine serum (Biowest, Riverside, MO, USA) and penicillin (100 U/mL)-streptomycin (100 μg/mL) (Nacalai-tesque) under 5% CO_2_ and 37°C according to a previous study [18]. Mycoplasma contamination was routinely tested by using the e-Myco^TM^ Mycoplasma PCR Detection Kit (iNtRON Biotechnology Inc., Korea), and the results were negative. Cells were subcultivated by culture incubation with 0.25% solution of trypsin-EDTA (Nacalai-tesque) at a split ratio of 1:2 to 1:5 every week. High-passage fibroblasts (25–35 passages) were confirmed to have a lower proliferative activity and were used as senescent cells. Low-passage fibroblasts (5–15 passages) were used as a young cell control. Before experiments, the NHDFs were seeded in 96-well or 6-well plates (Watson Co. Ltd., Tokyo, Japan), and then treated with dimethyl sulfoxide (DMSO; 1:1000, Nacalai-tesque) or puerarin (25–50 μM; product number P1886, lot number OK64L-NG; Dojindo, Kumamoto, Japan) and fulvestrant (1 μM; Wako Chemicals, Japan) [36] the following day, and were cultured for over 72 h. We used 25–50 μM puerarin in the culture medium since puerarin and a few other flavonoids have been used at 10–100 μM concentrations in previous studies [12, 35, 36].

### Cell viability and cell injury assays

NHDFs were seeded in 96-well plates at 2,500–10,000 cells per well and then cultured with puerarin. Cell viability and cell toxicity were evaluated using the Cell counting kit-8 assay (Cat No. 341–07624, Dojindo) and lactate dehydrogenase (LDH) assay kit (Cat No. 299–50601, Dojindo), respectively, according to the manufacturer’s protocols.

### Beta-galactosidase activity

The senescence-associated (SA) beta-galactosidase activity was detected as previously described [37]. In brief, the cells were fixed with 10% formalin (Nacalai-tesque) in phosphate-buffered saline (PBS) (Nacalai-tesque), and then incubated in X-gal solution (5 mM potassium ferricyanide, 5 mM potassium ferrocyanide, 2 mM MgCl_2_, and 0.5 mg/mL X-gal in citric acid/potassium phosphate buffer [pH 6.0]) at 37°C overnight. The number of stained cells per microscopic field of view (×100) was counted as senescent cells (Axiovert 200M, Zeiss, Oberkochen, Germany).

### Bromodeoxyuridine/5-bromo-2′-deoxyuridine (BrdU) assay

BrdU assays were performed as previously described [38] with brief modifications. The NHDFs were plated in 96-well plates at 5,000 cells per well. After culturing the cells with or without puerarin, BrdU (Nacalai-tesque) was added to the culture medium and the cells were cultured for a further 24 h. After fixation with 10% formalin in PBS and treatment with 2 M HCl for 10 min at room temperature (20 ~ 30°C) and for 20 min at 37°C to denature DNA, the BrdU incorporated in DNA was detected using a immunocytochemical method as described below. The number of BrdU-positive cells per microscopic field of view (×100) was calculated.

### Measurement of intracellular ROS

The cells were cultured in 96-well plates with or without puerarin for 72 h, followed by the addition of CellROX Green reagent (final concentration: 5 μM, Thermo Fisher Scientific, Waltham, MA, USA) to the culture medium and incubation at 37°C for 30 min, as described in manufacture’s protocol. Subsequently, the cells were washed and fixed with 10% formalin in PBS, and observed via fluorescence microscopy (LEICA DMi8, Leica, Wetzlar, Germany). The fluorescence intensity was determined by using the ImageJ software (version 1.53e, National Institutes of Health, Bethesda, MD, USA).

### mRNA expression analysis

Total RNA was isolated from the NHDFs using the FastGene™ RNA Basic Kit (Fast Gene, Japan) according to the manufacturer’s protocol. Single strand cDNA was generated from 0.1 μg of total RNA using the Rever Tra Ace kit (Toyobo, Osaka, Japan) according to the manufacturer’s protocol. Quantitative polymerase chain reaction (RT-PCR) analysis was performed using the Applied Biosystems Step One system (Applied Biosystems, Foster City, CA, USA) with the THUNDERBIRD SYBR qPCR Mix (Toyobo) according to the manufacturer’s protocols. The primers used are shown in Table 1. The following RT-PCR protocol was employed: initial activation step at 95°C for 1 min followed by 40 cycles of denaturation at 95°C for 15 s and annealing at 60°C for 1 min. All RT-PCR experiments were performed in duplicate. The accuracy of each reaction was confirmed from the difference in Ct value of the duplicates.

**Table 1:** Primers used for real-time PCR.

Target	Forward	Reverse
SMA	**ACTGAGCGTGGCTATTCCTTCGTT**	**GCAGTGGCCATCTCATTTTCA**
PDPN	**AAGAGCTGAAGGGTTACGCC**	**CACGGGTCATCTTCTCCCAC**
CNN1	**GGAAATTCGAGCCGGGGAA**	**TGGCAAACTTGTTGGTGCC**
GAPDH	**GTGGTCTCCTCTGACTTCAACA**	**GCTGTAGCCAAATTCGTTGTCA**

To consider the specificity of the primer set for SMA, a set of primers which has been designed to selectively detect the human SMA was used [39]. The primers for other genes were originally designed by using the Primer-BLAST tool (http://www.ncbi.nlm.nih.gov/tools/primer-blast/).

### Immunofluorescence microscopic examination

The NHDFs were fixed with 10% formalin in PBS and then permeabilized using 0.1% Triton-X 100 (Nacalai-tesque) in PBS for 10 min, and blocked in BlockingOne reagent (Nacalai-tesque). Immunostaining was performed using specific primary antibodies; the fluorescent secondary antibodies were diluted in the Can Get Signal Immunostain solution (Toyobo) according to a previous study [40]. The nuclei were stained with 4′,6-diamidino-2-phenylindole by using SlowFade Diamond Antifade Mountant with DAPI (Thermo Fisher Scientific) according to manufacture’s protocol. The cells were examined via fluorescence microscopy (LEICA DMi8, Leica) or using the Keyence BZ-X810 fluorescence microscope (Keyence, Japan). Antibodies used in this study were as follows: mouse anti-α-SMA IgG (ab7817, lot Number GR180235.1; Abcam, Cambridge, United Kingdom), mouse anti-calponin (CNN1) IgG (ZRB1146, lot number Q3250191; Sigma-Aldrich, St. Louis, MO, USA), rat anti-podoplanin (PDPN) IgG (B308013, lot number 337001; BioLegend, San Diego, CA, USA), mouse anti-estrogen receptor alpha (ERα IgG (ab16660, lot number GR3345665.5, Abcam), mouse anti-ERβ IgG (GTX70104, lot number 42142, GeneTex, Irvine, CA, USA), rat anti-BrdU IgG (ab6326, lot number GR91646.1, Abcam), CF488-conjugated anti-rat IgG (20023, lot number 17C0803, Biotium, Hayward, CA, USA), Alexa 488-conjugated goat anti-rabbit IgG (A11008, lot number 1622775; Thermo Fisher Scientific), Alexa 555-conjugated goat anti-mouse IgG (A21422, lot number 1990314, Thermo Fisher Scientific), Alexa 594-conjugated goat anti-mouse IgG (A11020, lot number 670014, Thermo Fisher Scientific). The fluorescence intensity was determined using the ImageJ software.

### Statistical analysis

The Student’s *t*-test (two-tailed) or *t*-test with Bonferroni correction (for multiple comparisons) was used to determine statistical significance between groups (Microsoft Excel software (2016)). Differences were considered significant with a P-value of <0.05.

## Results

### The protective effects of puerarin in senescent NHDFs

**Fig 1:**
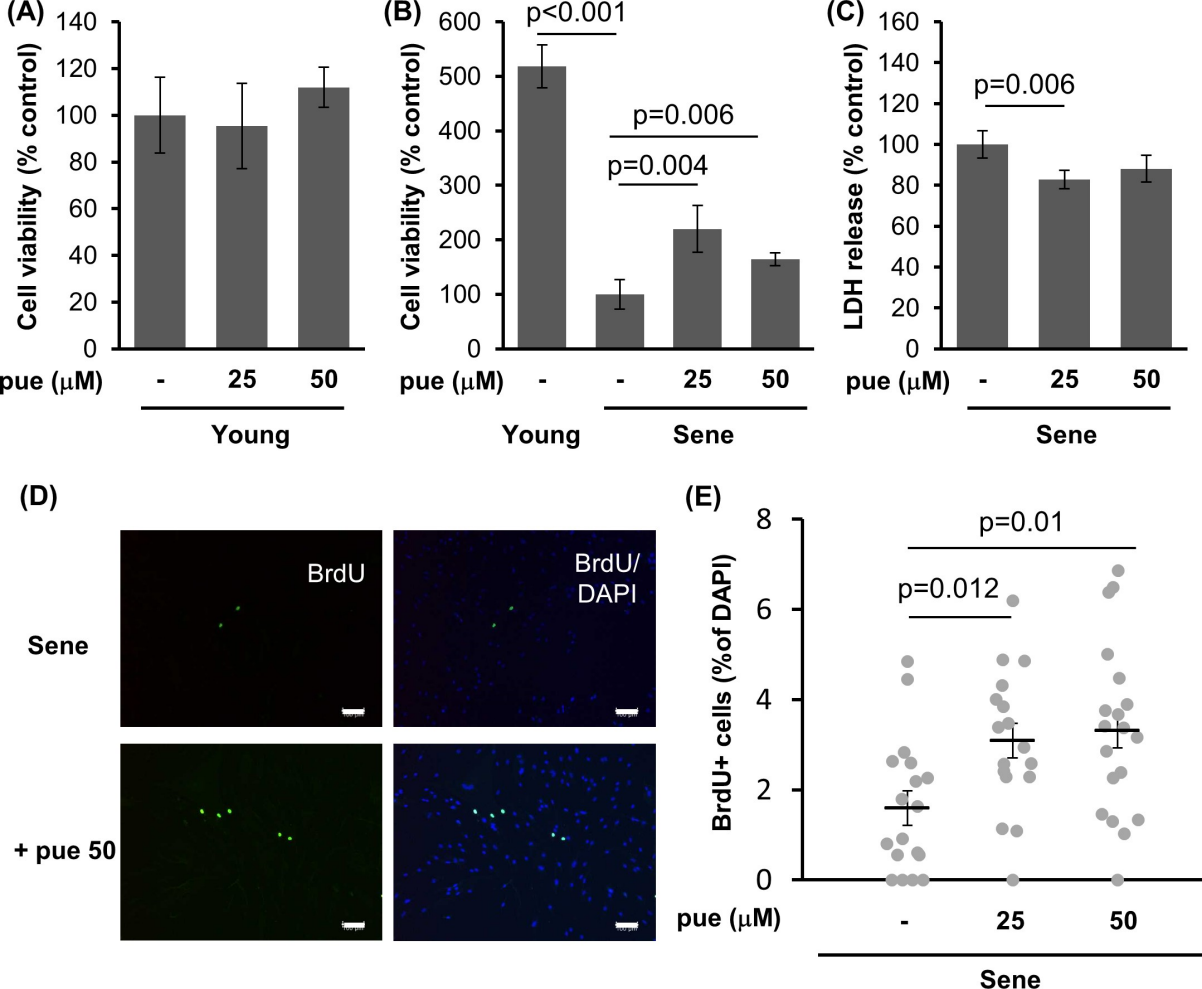
The effect of puerarin on the viability, injury and proliferative activity in high-passage NHDFs. A, B: The effect of puerarin (pue) on cell viability of young (A) and senescent NHDFs (B) was analyzed by CCK-8 assay. n = 5. C: The effect of cellular LDH release in high passage NHDFs. Data was obtained from LDH assay. n = 6. D, E: The effect of puerarin on the cell proliferative activity in senescent NHDFs. Cells were stained for BrdU and nuclei by specific antibody and DAPI, respectively, and photographed at 100×magnification (D). The percentage of BrdU positive cells per total cells was calculated from 15 microscopic fields for each group. (E). Scale bars = 100μm.

To determine the effects of puerarin on skin aging, we tested the effect of puerarin on cell viability in replication-induced senescence (Fig 1). After performing approximately 25–35 passages, the proliferation of NHDFs decreased compared to that of low-passage NHDFs. Treatment using puerarin (25 and 50 μM) for 72 h increased the cell viability of high-passage senescent NHDFs by 220% and 164%, respectively. However, it did not influence low-passage young NHDFs (Fig 1A and 1B). Cell injury during normal culturing, as measured via LDH release, was attenuated via puerarin treatment in senescent NHDFs (Fig 1C). In contrast, puerarin treatment did not influence the LDH release in young NHDFs (S1 Fig).

Next, we performed BrdU assays to investigate the effect of puerarin treatment on the proliferation rate in high-passage NHDFs (Fig 1D and 1E). BrdU-positive cells in microscopic fields were significantly increased in puerarin-treated NHDFs from 1.6% (control) to 3.1% (25 μM puerarin) and 3.3% (50 μM puerarin), indicating that puerarin treatment enhanced cell proliferation.

**Fig 2:**
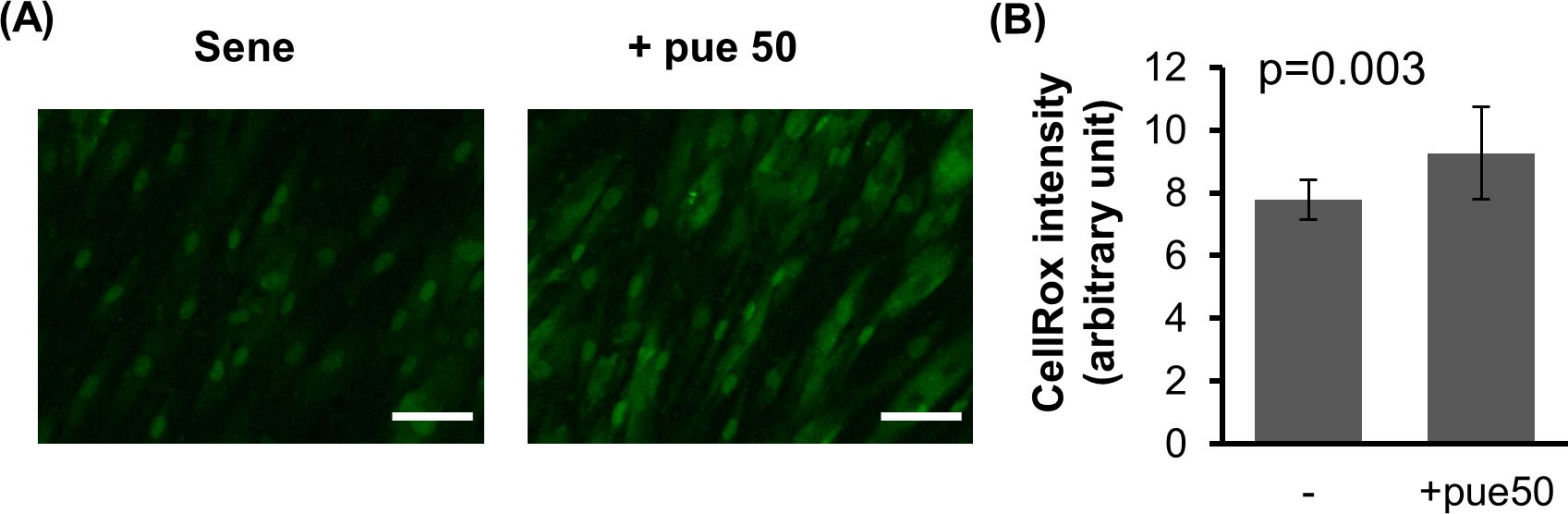
The effect of puerarin on ROS generation in high-passage NHDFs. ROS generation in senescent NFDFs (sene) with or without 50 μM puerarin (+pue 50) was measured by fluorescent ROS probe CellROX Green (A). CellROX intensity was calculated from 10 images for each group (B). Scale bars = 100μm.

We used ROS fluorescent probes to evaluate the effect of puerarin treatment on ROS generation in senescent NHDFs (Fig 2). Since isoflavones show anti-oxidant activity [36], we expected that puerarin treatment could reduce ROS production. However, puerarin treatment increased ROS production in senescent NHDFs. The ROS increase via puerarin treatment was not observed in young NHDFs (S2 Fig).

### The anti-aging effects of puerarin treatment in senescent NHDFs

**Fig 3:**
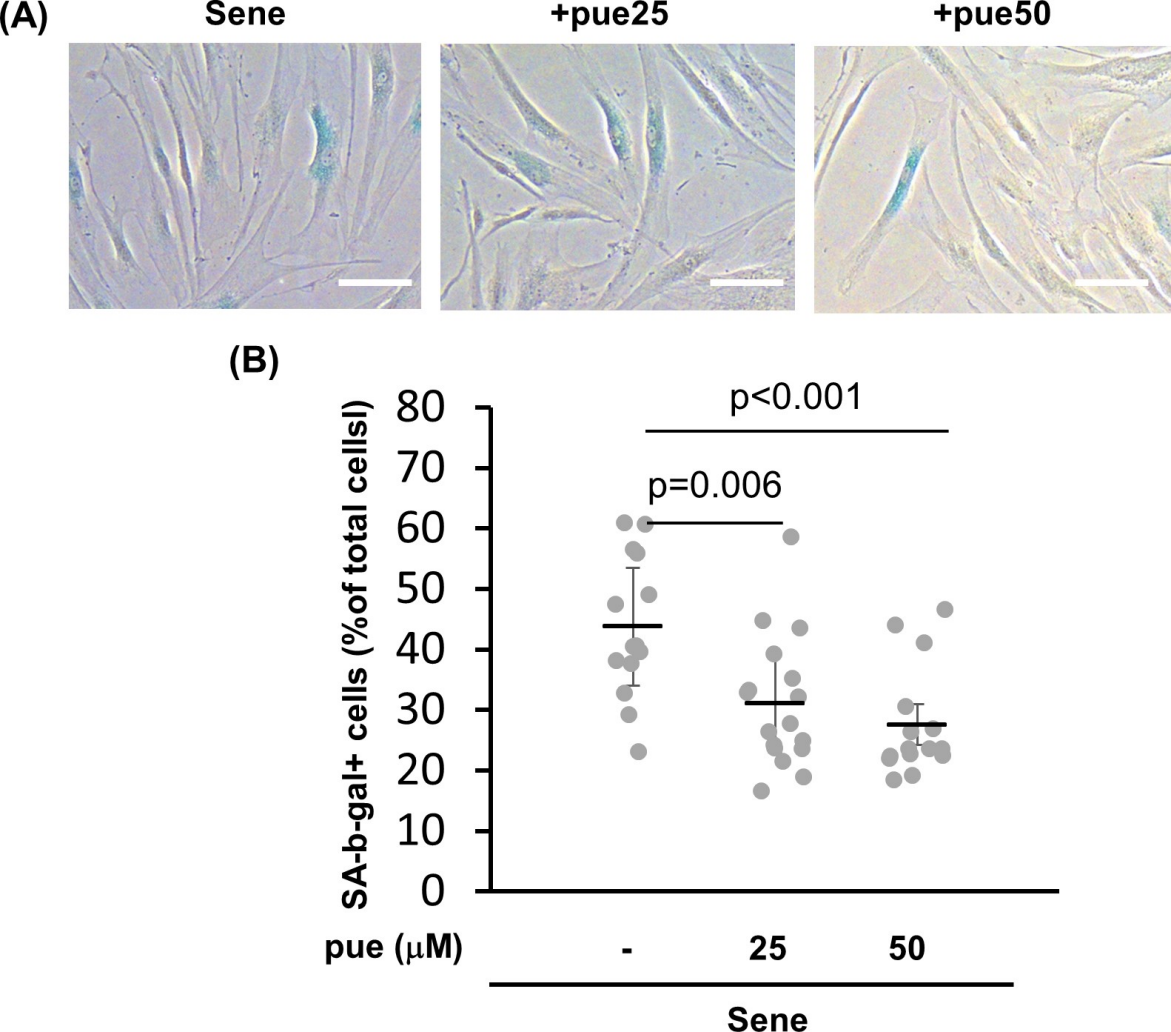
The effect of puerarin on cellular senescence in high-passage NHDFs. A, B: The effect of puerarin on SA-β-galactosidase activity. Young and senescent (sene) NHDFs treated with 20 or 50 μM puerarin (pue) were stained by SA-β-galactosidase assay (A). The percentage of SA-β-galactosidase positive cells per total cells was calculated from 15 microscopic fields for each group. Scale bars = 100 μm.

We evaluated the effect of puerarin treatment on the senescent phenotype of NHDFs. SA-beta-galactosidase-positive NDHFs decreased in proportion via puerarin treatment from 39% to 27% (25 μM puerarin) and 23% (50 μM puerarin) (Fig 3A and 3B).

**Fig 4:**
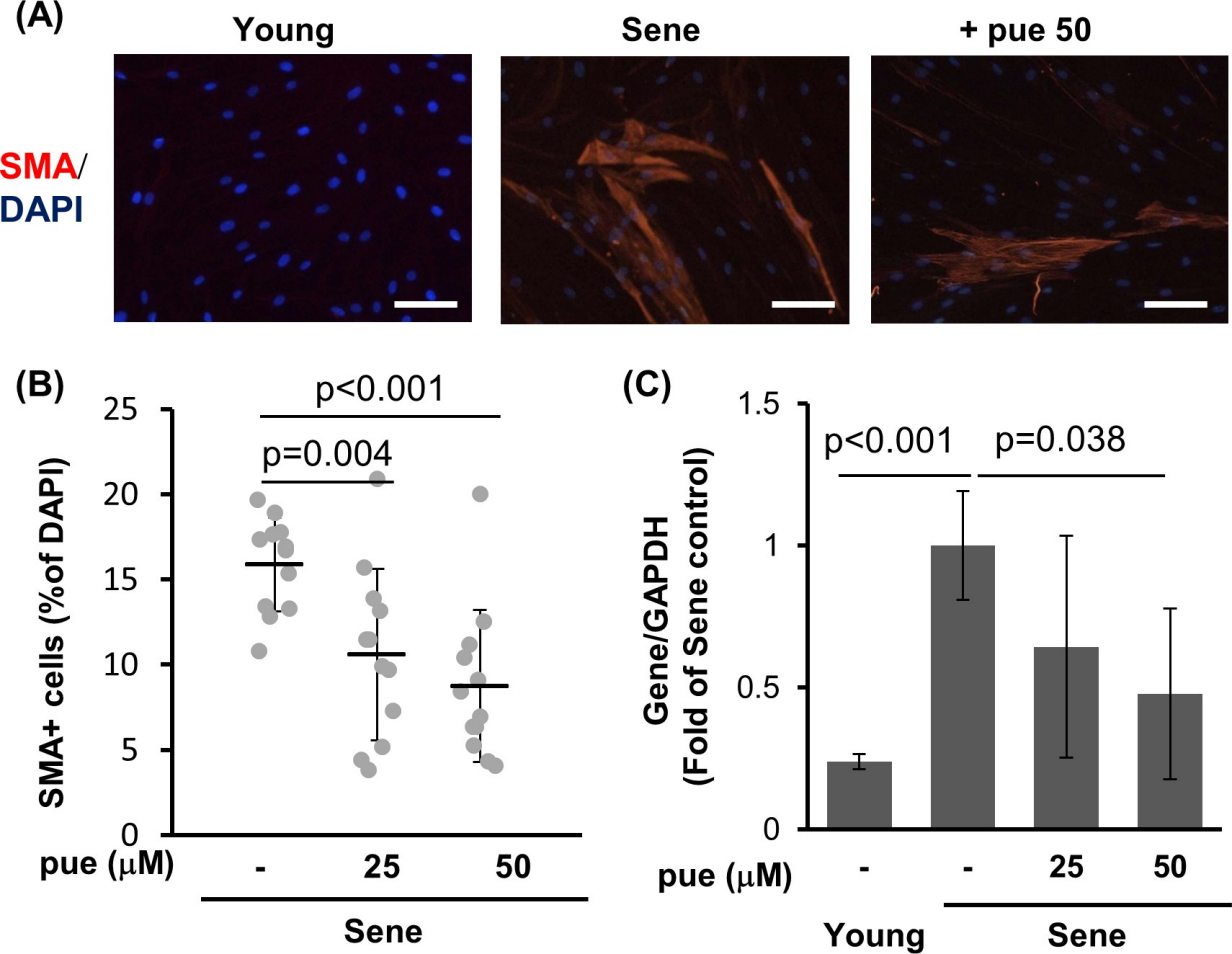
The effect of puerarin on the expression of SMA in high passage NHDFs. A-B: Young or senescent (sene) NHDFs with or without 50 μM puerarin (+pue 50) were stained for SMA by the immunocytochemical method. The percentage of SMA positive cells per total cells was calculated from 15 microscope fields for each group. Scale bars = 100μm. C The expression of SMA mRNA in young and senescent NHDFs treated with or without 25–50 μM puerarin (pue) for 72 h. n = 4.

A characteristic of aged dermal fibroblasts involves the decrease in papillary fibroblasts and an increase in reticular fibroblasts in the skin [5, 19]. In the NHDF culture model, the increased passaging of NHDFs leads to an increase in the number of cells showing reticular phenotype and SMA-positive myofibroblasts [20, 41]. Therefore, we evaluated the effect of puerarin treatment on the proportion of α-SMA-positive cells. Puerarin treatment reduced the number of α-SMA-positive cells in high-passage senescent NHDFs from 16% to 11% (25 μM puerarin) and 8.9% (50 μM puerarin) (Fig 4A). A reduction in α-SMA levels via puerarin treatment was also validated by analyzing mRNA expression; reduction of mRNA expression by 64 and 47% by treatment with 25 and 50 μM puerarin, respectively, was observed (Fig 4B).

**Fig 5:**
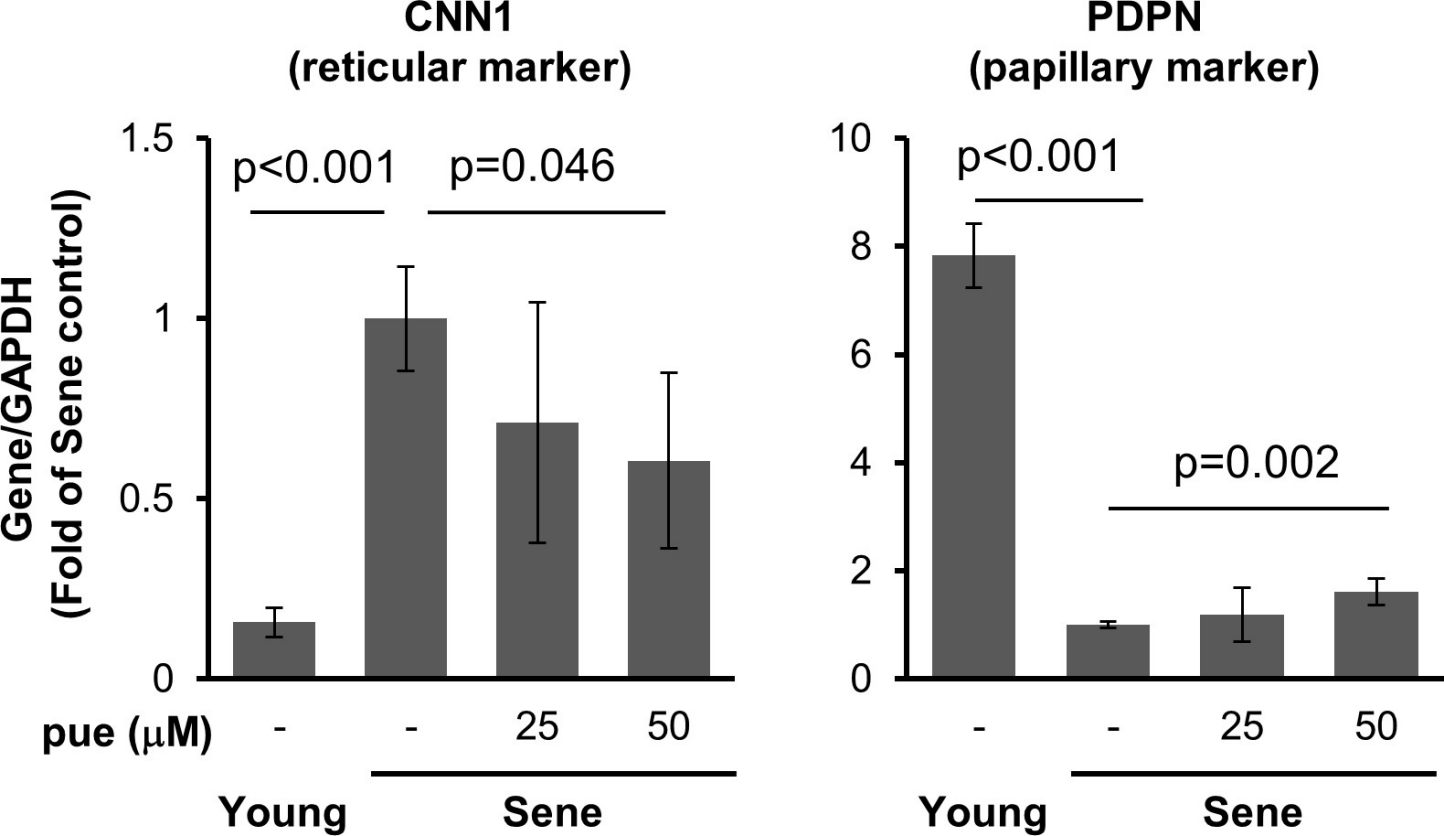
The effect of puerarin on gene expressions of reticular and papillary markers. The expressions of PDPN (papillary marker), CNN1 (reticular marker) in young and senescent NHDFs treated with or without 25–50 μM puerarin (pue) for 72 h. n = 3–5.

Next, we evaluated the effect of puerarin treatment on the expression of gene markers of papillary and reticular phenotypes, PDPN and CNN1, respectively (Fig 5A). Puerarin treatment upregulated PDPN in senescent NHDFs. In contrast, puerarin treatment reduced CNN1 expression in senescent NHDFs. CNN1 expression was increased in high-passage NHDFs compared to that in low passage NHDFs.

**Fig 6:**
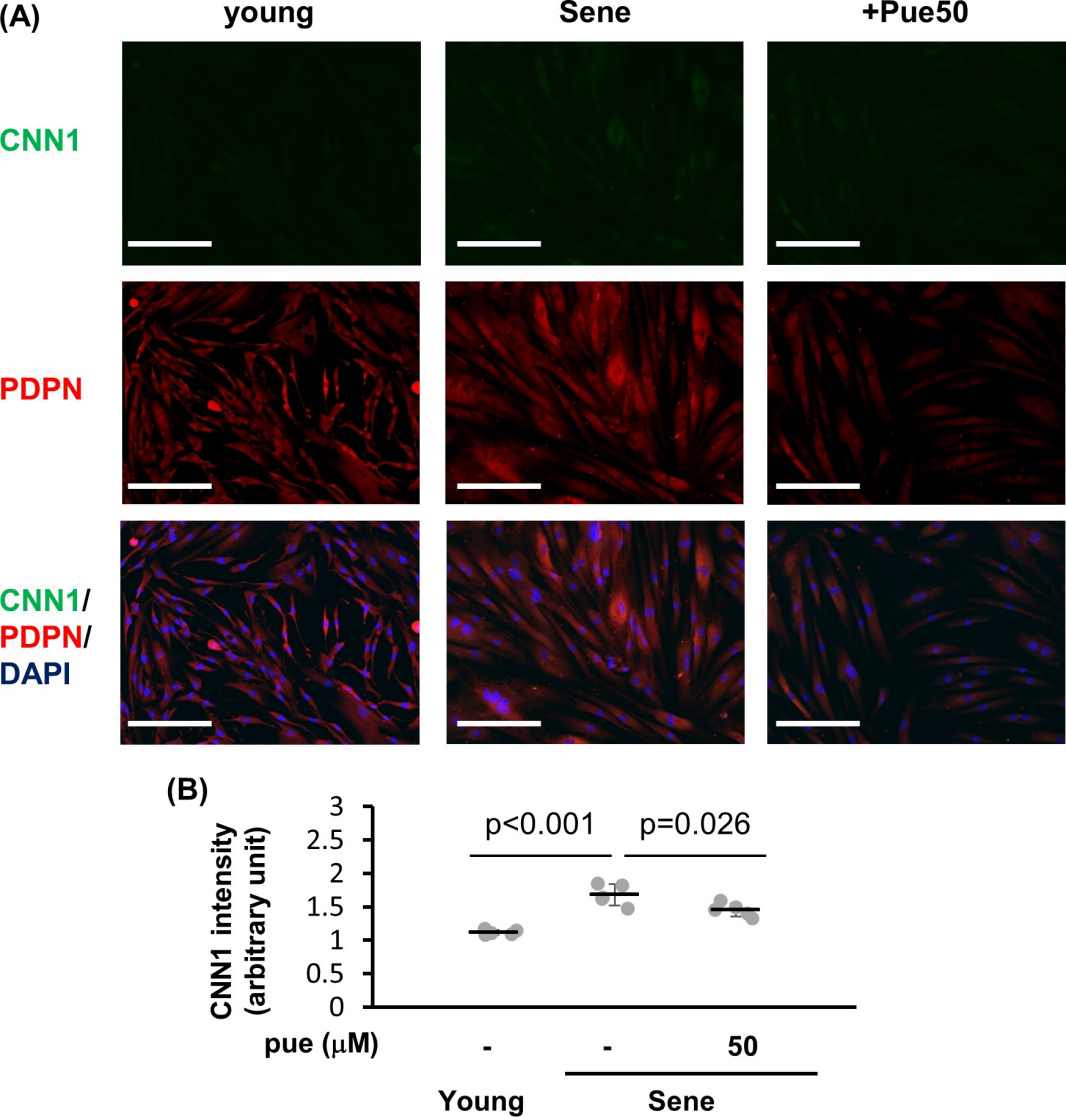
The effect of puerarin on CNN1 and PDPN expressions in senescent NHDFs. (A) Young or senescent (sene) NHDFs with or without 50 μM puerarin (+pue 50) were stained for CNN1 (green) and PDPN (red) by the immunocytochemical method. (B) Fluorescent intensities of CNN1 positive area were calculated from 5 microscpic fields for each group. Scale bars = 100μm.

We further evaluated the protein levels of PDPN and CNN1 via immunocytochemistry. Consistent with mRNA data, CNN1 intensity was higher in high-passage NHDFs than in low-passage NHDFs (Fig 6), indicating that high-passage NHDFs might have acquired a reticular disposition. Puerarin treatment reduced the CNN intensity by 87% in high-passage NHDFs indicating that puerarin might have prevented the transdifferentiation to reticular fibroblasts.

However, the PDPN intensity was similar between high-passage and low-passage NHDFs. All NHDFs were positive for PDPN antibodies, indicating that the high-passage NHDFs might have still possessed the papillary disposition. Puerarin treatment did not affect the PDPN intensity in NHDFs.

### The role of estrogen receptor signaling pathway in the anti-aging effects of puerarin treatment

**Fig 7:**
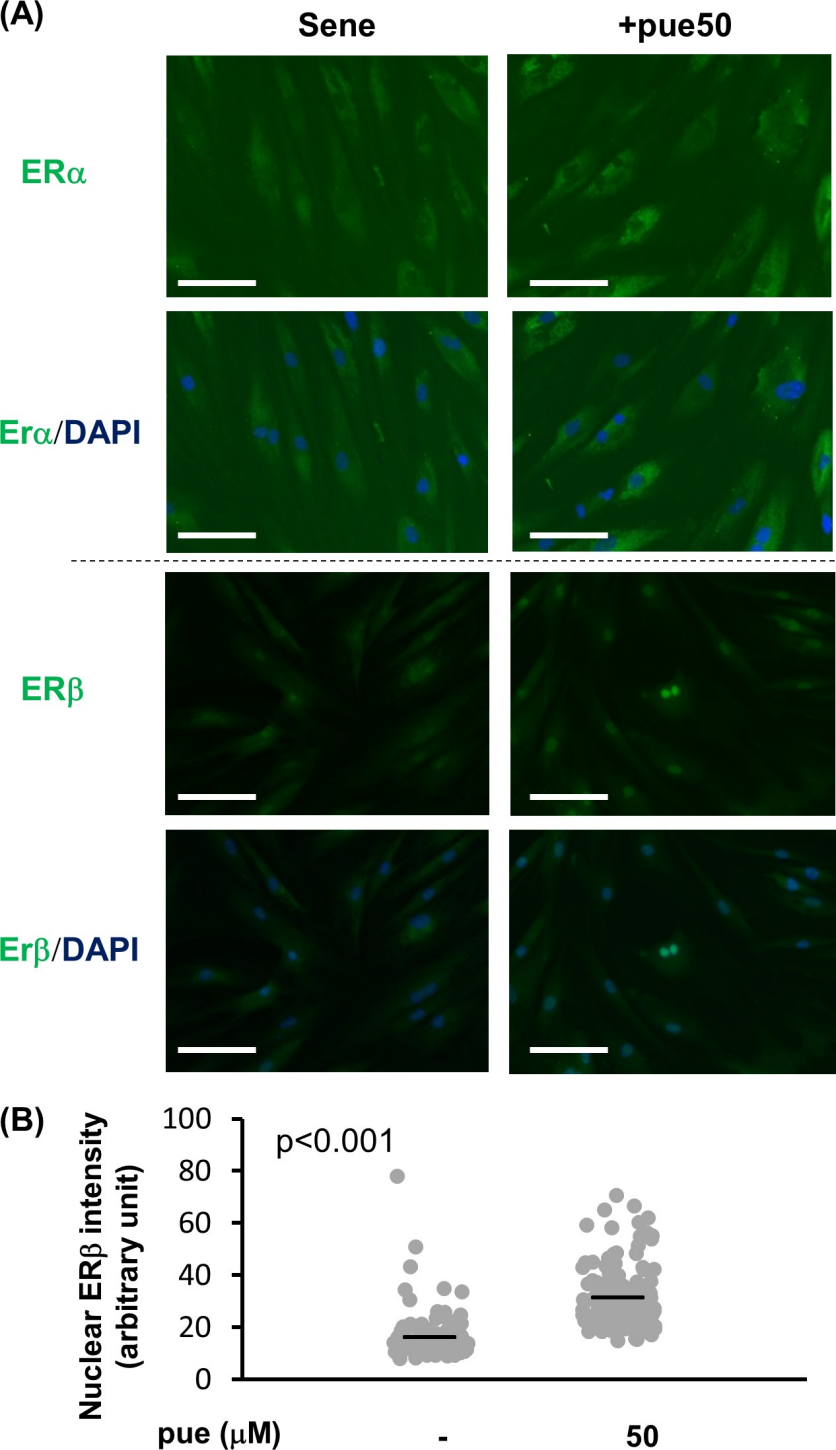
The effect of puerarin on the translocation of estrogen receptors in senescent NHDFs. (A) Senescent (sene) NHDFs with or without 50 μM puerarin (+pue 50) were stained for ERα (upper) or ERβ (lower) by the immunocytochemical method. (B) Fluorescent intensities of ERβ that translocated into nuclei were calculated from more than 100 nuclei (3 microscpic fields) for each group. Scale bars = 50μm.

Since isoflavones possess an ER agonist property, we investigated whether the ER pathways were involved in the effect of puerarin treatment on NHDFs. Firstly, we confirmed the effect of puerarin treatment on the localization of ERs via immunocytochemistry (Fig 7). ERα was not localized in the nuclei of control NHDFs; the intracellular location of ERα was not altered via puerarin treatment.

In contrast, the nuclear translocation of ERβ was enhanced via puerarin treatment. The nuclear intensity of ERβ was increased in puerarin-treated cells (Fig 7B). Additionally, the increase in nuclear ERβ was observed in low-passage NHDFs (S3 Fig). These data indicated that puerarin treatment activated ERβ in both high- and low-passage NHDFs.

**Fig 8:**
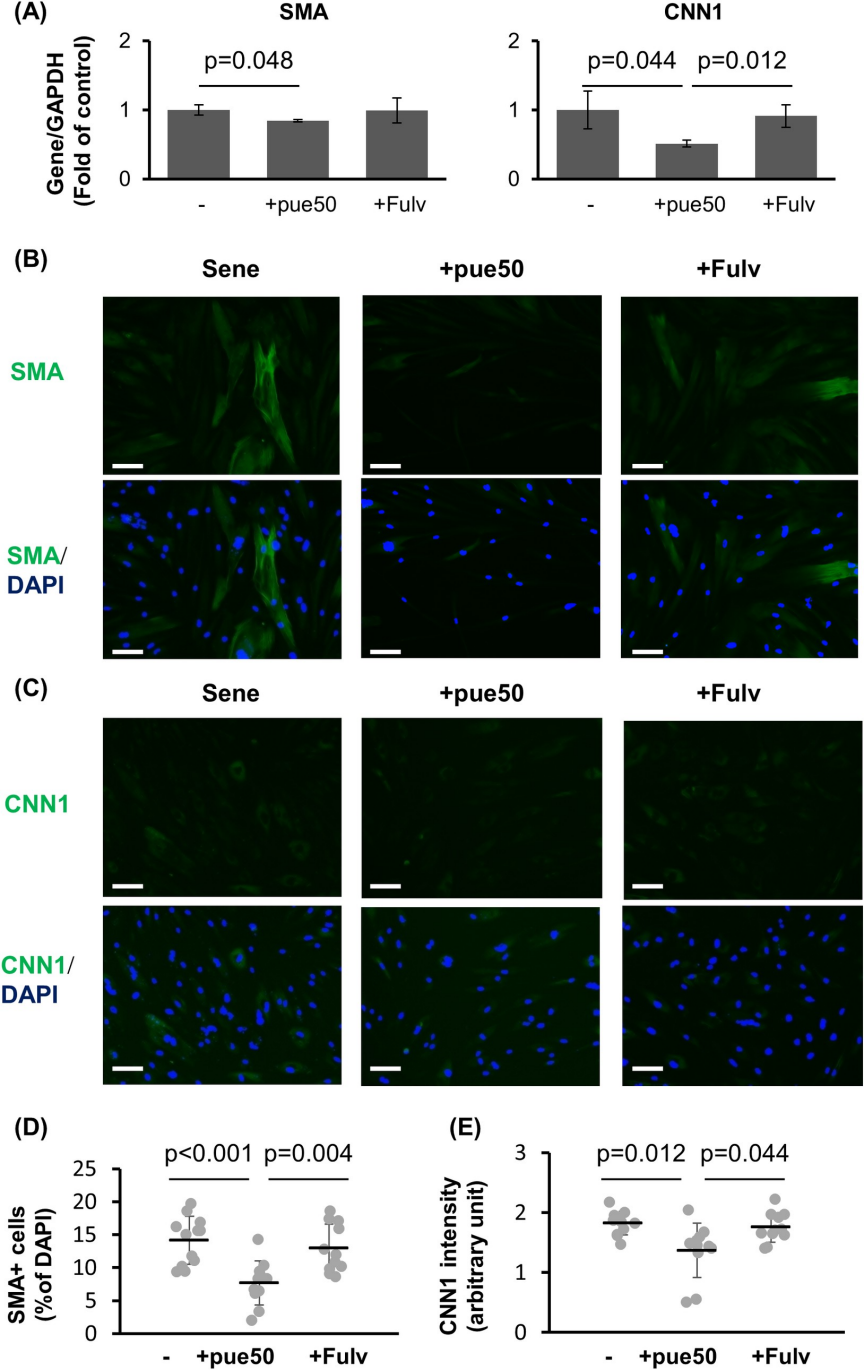
Estrogen receptor antagonist blocked puerarin-mediated reduction of SMA and CNN1. (A) The expressions of SMA and CNN1 mRNAs in senescent NHDFs treated with 50 μM puerarin (+pue50) and estrogen receptor antagonist, 1 μM fulvostrant (+Fulv) for 72 h. n = 4. (B-E) Senescent (sene) NHDFs treated with 50 μM puerarin (+pue50) and estrogen receptor antagonist, 1 μM fulvostrant (+Fulv) were stained for SMA (B) and CCN1 (C) by the immunocytochemical method. (D) The percentage of SMA positive cells per total cells was calculated from 11 microscope fields for each group. (E) Fluorescent intensities of CNN1 positive area were calculated from 11 microscpic fields for each group. Scale bars = 100μm.

We analyzed the role of ERs in the anti-aging activity of puerarin in NHDFs. Fulvestrant, an ER antagonist, blocked the puerarin-induced downregulation of SMA and CNN1 (Fig 8A). Furthermore, immunocytochemical analysis showed that fulvestrant treatment blocked the effect of puerarin on SMA-positive cells (Fig 8B and 8D). The reduction of CNN1 via puerarin treatment was also blocked by fulvestrant treatment (Fig 8C and 8E). These data indicate that the attenuation of transdifferentiation to reticular fibroblasts via puerarin treatment might have been associated with the ER signaling pathway.

## Discussion

In the present study, we evaluated the effects of puerarin treatment on senescent NHDFs and discovered that puerarin treatment enhanced the viability of NHDFs, accompanied by an increased proliferative activity and a decreased cell injury. Furthermore, we found that puerarin treatment lowered the number of SA-beta-galactosidase-positive cells. Puerarin treatment also attenuated the aging-related fibroblast subpopulation changes; a decrease in the number of myofibroblasts and reticular fibroblasts via puerarin treatment was observed. Taken together, puerarin treatment may prevent age-related fibroblast transdifferentiation.

An increased passaging did not affect the proportion of PDPN-positive cells, a marker of papillary fibroblasts, whereas the PDPN mRNA expression was lower in high-passage NHDFs than in low-passage NHDFs. These data indicate that the transdifferentiated NHDFs may still have a papillary disposition. To determine how puerarin influences the papillary disposition in NHDFs, other cell markers should be analyzed.

Puerarin possesses a wide variety of pharmacological properties, and their various molecular mechanisms have been proposed [29]. Among them, puerarin is recognized as a phytoestrogen. Puerarin has beneficial effects against menopausal symptoms such as bone loss [25]. It has been reported that puerarin shows estrogenic activity via ERβ in various types of cells [25, 29, 42]. Consistent with previous reports, we also confirmed that puerarin treatment increased the translocation of ERβ and not of ERα in NHDFs. Moreover, we found that the ER-inhibitor fulvestrant blocked the effect of puerarin on SMA and CNN1 expression, indicating that the effects of puerarin on senescent NHDFs may be associated with the ER pathway. Estrogens are key hormones in skin aging homeostasis and act directly on dermal fibroblasts [43, 44]. ERβ activation by genistein, a selective ERβ activator, increases cell viability in hydrogen peroxide-treated NHDFs concomitant with the modulation of mitochondrial membrane potential [45]. ERβ-selective ligands, WAY-200070 and ERB-041, suppress the expression of matrix metalloproteases and cytokines induced by UV irradiation in NHDFs [46], indicating the role of ERβ in collagen synthesis. Therefore, ERβ activation by puerarin may block various types of cellular aging processes, such as oxidative stress-induced aging and UV-induced photoaging. Moreover, ERs can activate multiple signaling pathways, such as mitogen-activated protein kinases (ERK1/2 and p38) and the phosphoinositide 3-kinase (PI3K)/AKT pathway [47]. It has been reported that few of the cytoprotective roles of puerarin are mediated via Akt activation [36, 48, 49]. Notably, Hwang et al. demonstrated that puerarin-mediated protection against oxidative stress is associated with the ER-dependent PI3K/Akt-Nrf2 signaling pathway in hepatic cells [36]. Therefore, the ERβ/Akt signaling pathway may be involved in the protective effect of puerarin against cellular senescence in NHDFs. Further studies are needed to elucidate the mechanism in NHDFs.

Puerarin has been shown to possess antioxidant properties [29]. In the present study, we found that ROS production was not decreased, but rather increased in puerarin-treated senescent NHDFs. In the case of proliferation-induced senescent cells, excessive ROS production may not be associated with cellular defects. In contrast, an appropriate generation of ROS has beneficial anti-aging roles by modulating certain signaling pathways such as STE-like protein kinase and Jun N-terminus kinase signaling pathways [2]. It is also known that an appropriate level of oxidative stress is necessary for cell proliferation in certain types of cells, including T cells and cancer cells [2]. These facts raise the possibility that the appropriate generation of oxidative stress may be related to puerarin-induced cell proliferation. It has been reported that puerarin treatment restores mitochondrial function in the skeletal muscle in diabetic rats [50]. Since mitochondrial activity is a major source of ROS production, activation of the mitochondrial function is one potential pathway connecting ROS production with puerarin.

Senolytics, such as quercetin and dazatinib, induce cell death preferentially in senescent cells [12]. Quercetin treatment decreases cell viability and increases cell death in senescent preadipocytes, but not in non-senescent cells. We expected that puerarin also possessed senolytic properties. However, we observed that puerarin treatment did not induce cell death or toxicity in high-passage NHDFs, as confirmed via LDH assays (Fig 1C) and ethidium homodimer-I assay (live-dead assay, S4 Fig), whereas we observed a reduction in the proportion of SA-beta-galactosidase-positive cells after puerarin treatment. Taken together, puerarin may not be considered as a senolytic agent for senescent NHDFs.

Our present study indicates that a *P*. *lobata* diet may confer anti-aging properties to the skin. The bioavailability of orally ingested puerarin in rodents is approximately 4–7% [34]. When 10 mg/kg body weight of puerarin is administered to rodents, the maximal blood concentration of puerarin is 228 μg/L (approximately 0.5 μM). This concentration is lower than the puerarin concentration used for cell treatment in this study. To confirm the effects of *P*. *lobata* diet on skin aging, the clinical effects must be evaluated in the future.

## Supporting information

S1 Fig The effect of puerarin on cell toxicity in low-passage NHDFs. The effect of cellular LDH release in low passage NHDFs. Data was obtained from LDH assay. n = 6.
(PPTX)

S2 Fig The effect of puerarin on ROS generation in low-passage NHDFs. ROS generation in young NFDFs (Young) with or without 50 microM puerarin (+pue 50) was visualized by fluorescent ROS probe CellROX Green.
(PPTX)

S3 Fig The effect of puerarin on the translocation of estrogen receptors in young NHDFs. Young NHDFs with or without 50 microM puerarin (+pue 50) were stained for ER#a (upper) or ER#b (lower) by the immunocytochemical method.
(PPTX)

S4 Fig The effect of puerarin on cell death in senescent NHDFs. Dead cells and live cells were stained by ROS generation in young NFDFs (Young) with or without 50 microM puerarin (+pue 50) was visualized by fluorescent probes Ethydium Homodimer-1 and calcein, respectively.
(PPTX)

S1 Dataset (PDF)

## Abbreviations

NHDFs: normal human dermal fibroblasts
SMA: smooth muscle actin
CNN1: calponin 1
ER: estrogen receptor
