# Supplementary material for: Puerarin blocks the aging phenotype in human dermal fibroblasts
Source: PLoS One. 2021 Apr 22;16(4):e0249367. doi: 10.1371/journal.pone.0249367 (PMC8061915; doi:10.1371/journal.pone.0249367)
Supplement: S1 Fig — The effect of cellular LDH release in low passage NHDFs. Data was obtained from LDH assay. n = 6. (PPTX) [file pone.0249367.s001.pptx]

## Slide 1
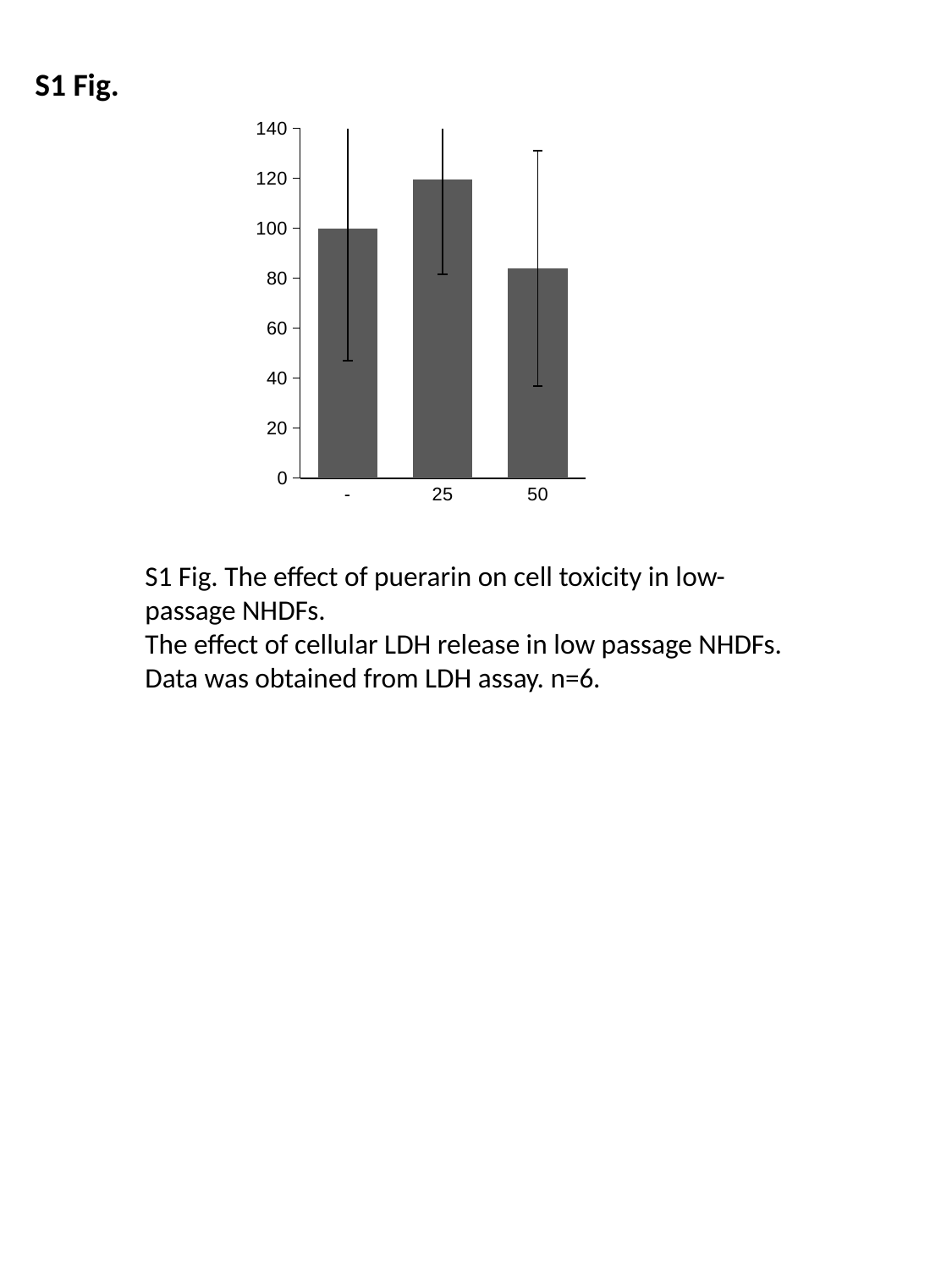

S1 Fig.
### Chart
| Category | |
|---|---|
| - | 100.0 |
| 25 | 119.40839118623607 |
| 50 | 84.00241472985213 |S1 Fig. The effect of puerarin on cell toxicity in low-passage NHDFs.
The effect of cellular LDH release in low passage NHDFs. Data was obtained from LDH assay. n=6.
