# Supplementary material for: Puerarin blocks the aging phenotype in human dermal fibroblasts
Source: PLoS One. 2021 Apr 22;16(4):e0249367. doi: 10.1371/journal.pone.0249367 (PMC8061915; doi:10.1371/journal.pone.0249367)
Supplement: S2 Fig — ROS generation in young NFDFs (Young) with or without 50 microM puerarin (+pue 50) was visualized by fluorescent ROS probe CellROX Green. (PPTX) [file pone.0249367.s002.pptx]

## Slide 1
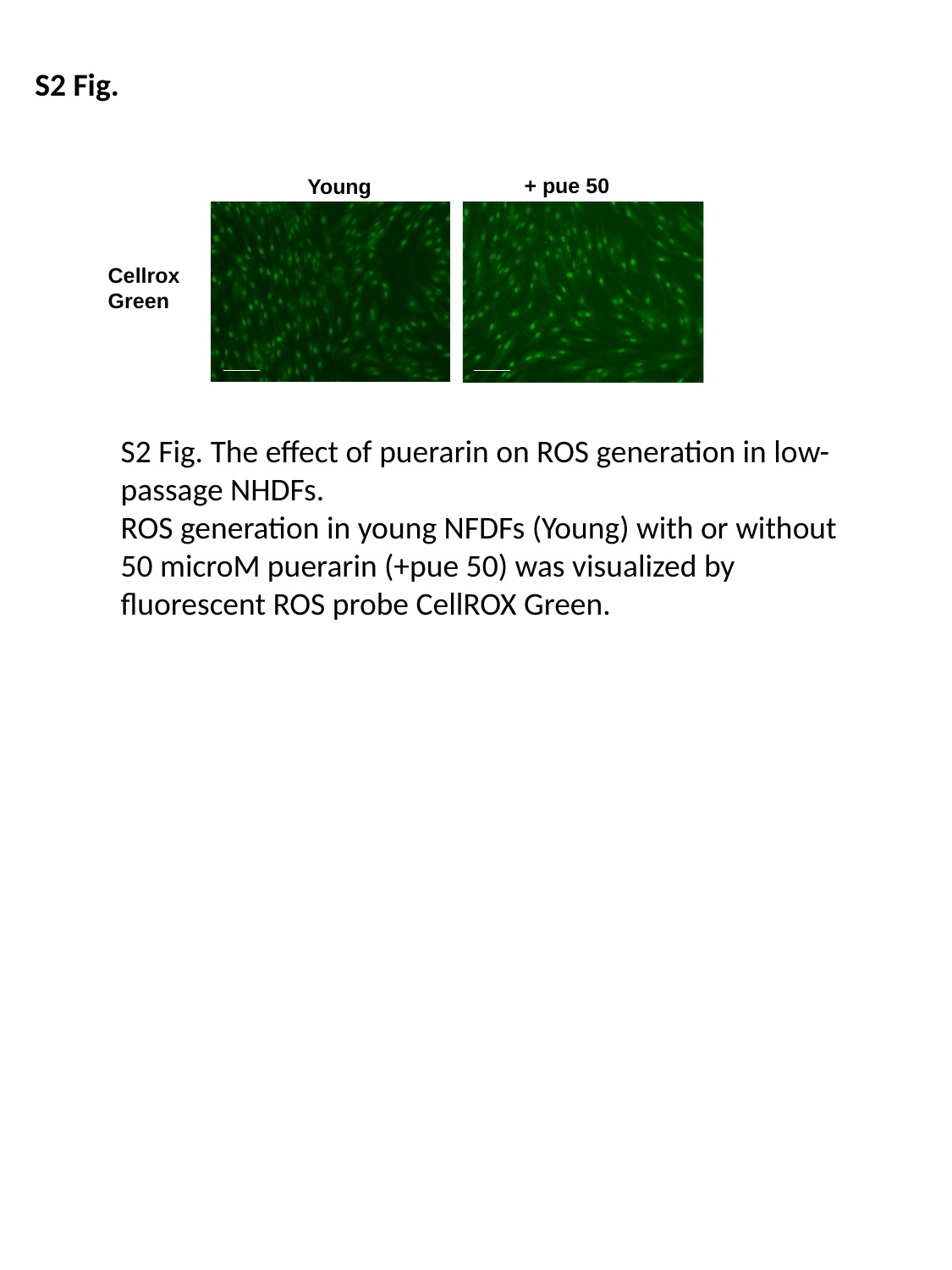

S2 Fig.
+ pue 50
Young
Cellrox
Green
S2 Fig. The effect of puerarin on ROS generation in low-passage NHDFs.
ROS generation in young NFDFs (Young) with or without 50 microM puerarin (+pue 50) was visualized by fluorescent ROS probe CellROX Green.
