# Supplementary material for: Puerarin blocks the aging phenotype in human dermal fibroblasts
Source: PLoS One. 2021 Apr 22;16(4):e0249367. doi: 10.1371/journal.pone.0249367 (PMC8061915; doi:10.1371/journal.pone.0249367)
Supplement: S3 Fig — Young NHDFs with or without 50 microM puerarin (+pue 50) were stained for ER#a (upper) or ER#b (lower) by the immunocytochemical method. (PPTX) [file pone.0249367.s003.pptx]

## Slide 1
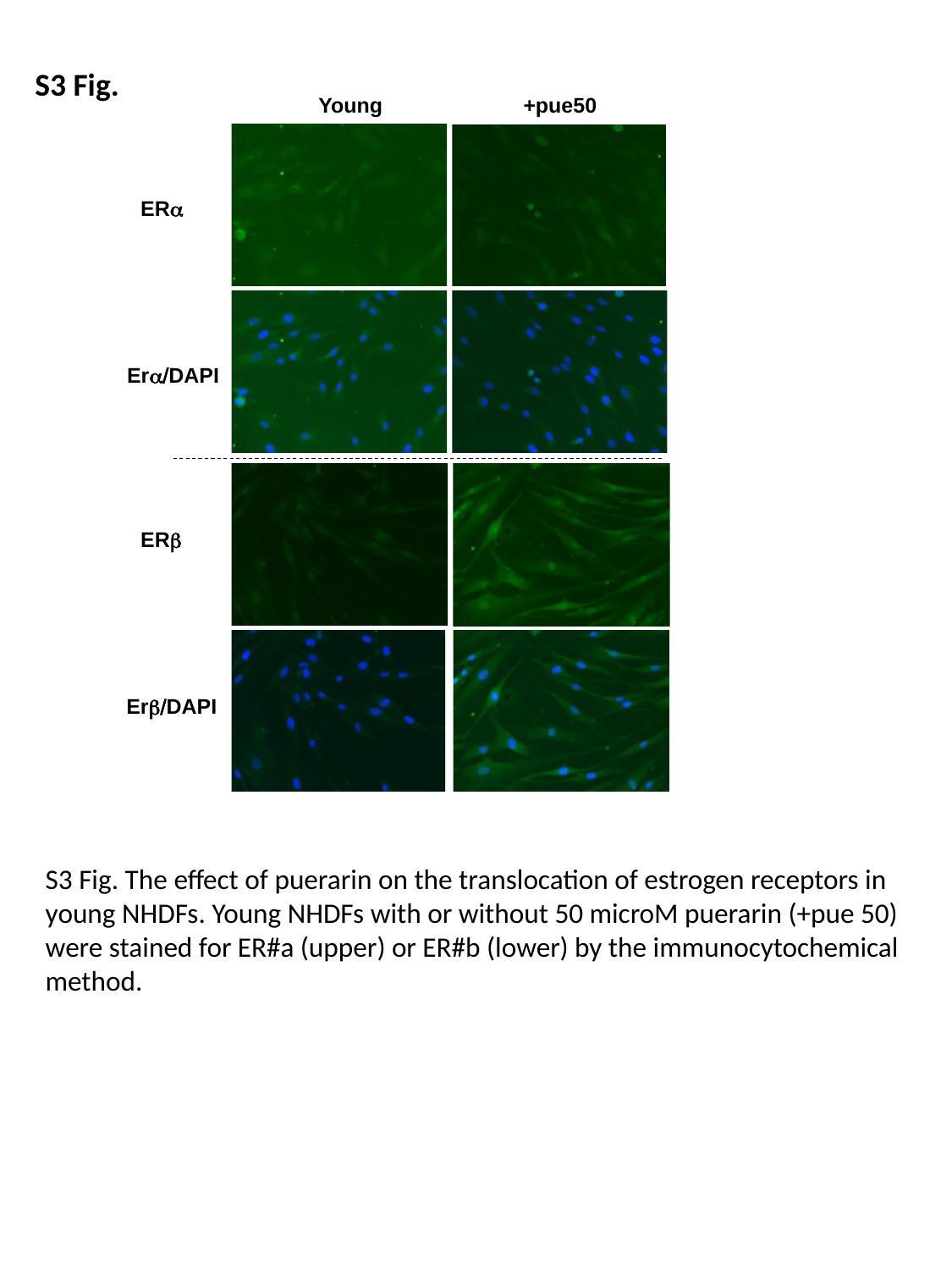

S3 Fig.
Young
+pue50
ERa
Era/DAPI
ERb
Erb/DAPI
S3 Fig. The effect of puerarin on the translocation of estrogen receptors in young NHDFs. Young NHDFs with or without 50 microM puerarin (+pue 50) were stained for ER#a (upper) or ER#b (lower) by the immunocytochemical method.
