# Supplementary material for: Puerarin blocks the aging phenotype in human dermal fibroblasts
Source: PLoS One. 2021 Apr 22;16(4):e0249367. doi: 10.1371/journal.pone.0249367 (PMC8061915; doi:10.1371/journal.pone.0249367)
Supplement: S4 Fig — Dead cells and live cells were stained by ROS generation in young NFDFs (Young) with or without 50 microM puerarin (+pue 50) was visualized by fluorescent probes Ethydium Homodimer-1 and calcein, respectively. (PPTX) [file pone.0249367.s004.pptx]

## Slide 1
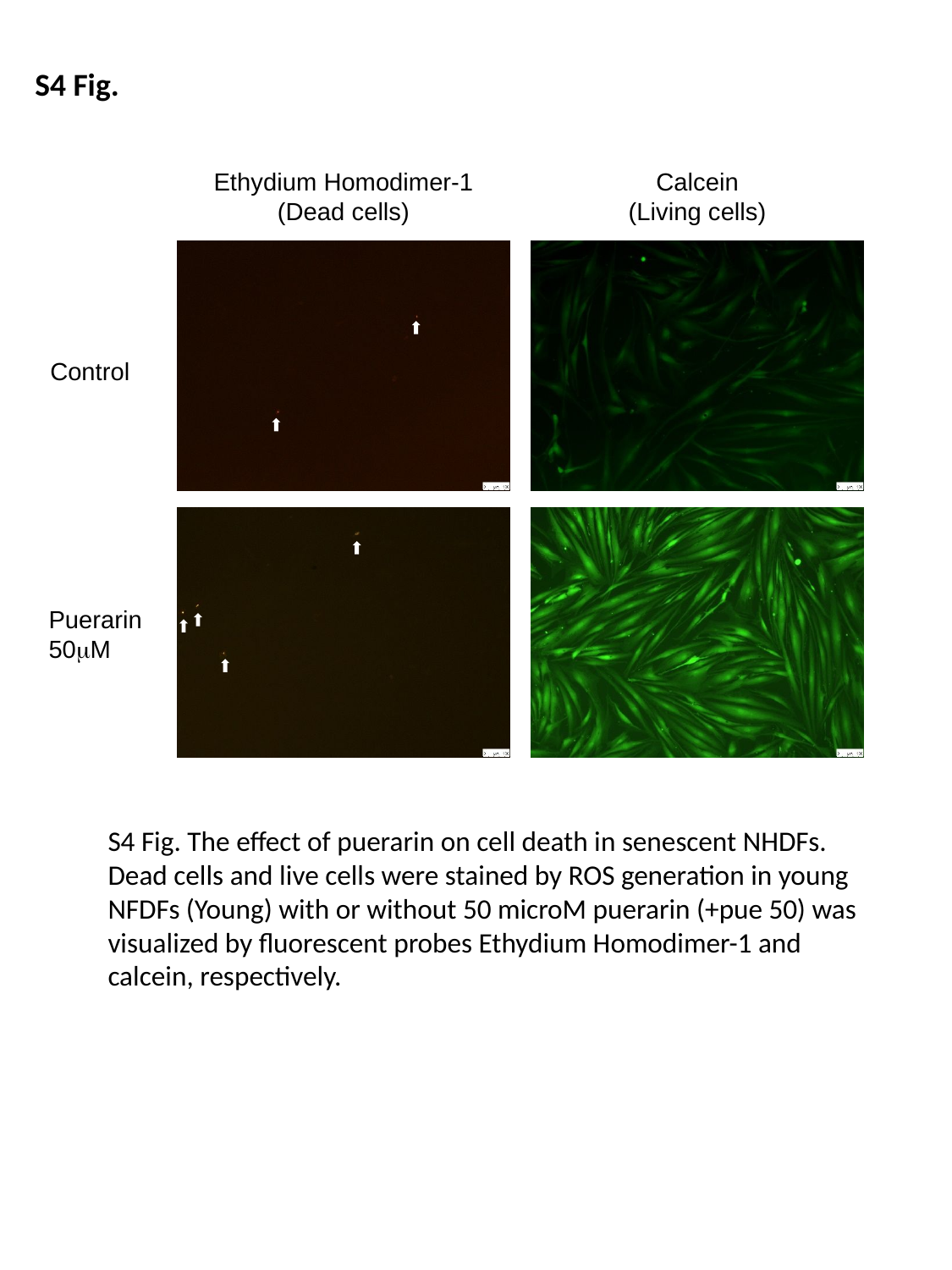

S4 Fig.
Ethydium Homodimer-1
(Dead cells)
Calcein
(Living cells)
Control
Puerarin
50mM
S4 Fig. The effect of puerarin on cell death in senescent NHDFs.
Dead cells and live cells were stained by ROS generation in young NFDFs (Young) with or without 50 microM puerarin (+pue 50) was visualized by fluorescent probes Ethydium Homodimer-1 and calcein, respectively.
