# Supplementary material for: Puerarin blocks the aging phenotype in human dermal fibroblasts
Source: PLoS One. 2021 Apr 22;16(4):e0249367. doi: 10.1371/journal.pone.0249367 (PMC8061915; doi:10.1371/journal.pone.0249367)
Supplement: S1 Dataset — (PDF) [file pone.0249367.s005.pdf]

S1 Data set.

Fig. #	Mean	S.D	Statistical method used	P value	# samples
**Fig. 1A**			Bonferroni: compare selected pairs		
Control	100	16.16042423		-	5
Pue25	95.35572513	18.32464891		-	5
Pue50	111.9438902	8.644263465		-	5
**Fig, 1B**			Bonferroni: compare selected pairs		
Young	518.5139202	39.46213987		P<0.001 vs Sene-Control	5
Sene-Control	100	26.85378475			5
Sene-Pue25	220.1326185	43.4212541		P<0.001 vs Sene-Control	5
Sene-Pue50	164.4610609	11.45393153		P<0.001 vs Sene-Control	5
**Fig. 1C**			Bonferroni: compare selected pairs		
Sene-Control	100	6.757593926			4
Sene-Pue25	82.81375161	4.487181989		P=0.006 vs Sene-Control	4
Sene-Pue50	88.13980481	6.446533514			4
**Fig. 1E**			Bonferroni: compare selected pairs		
Sene-Control	1.59167094	1.518551808			18
Sene-Pue25	3.091727801	1.556556173		P=0.005	18
Sene-Pue50	3.315534162	1.997252115		P=0.005	18
**Fig. 2B**			Student t-test		
Control	7.774692308	0.628574099			13
Pue50	9.268333333	1.469817696		P=0.003	12
**Fig. 3B**			Bonferroni: compare selected pairs		
Sene-Control	43.79073414	11.78395576			10
Sene-Pue25	31.06200268	10.81345626		P=0.006	10
Sene-Pue50	27.60165488	9.016801532		P<0.001	10
**Fig. 4B**			Bonferroni: compare selected pairs		
Sene-Control	15.89257914	2.745589679			12
Sene-Pue25	10.5839117	5.017861591		P=0.003	12
Sene-Pue50	8.749712695	4.449577841		P<0.001	12
**Fig. 4C**			Bonferroni: compare selected pairs		
Young	0.23832756	0.027075225			3
Sene-Control	1	0.192147918			5
Sene-Pue25	0.643660751	0.391113283			5
Sene-Pue50	0.477387362	0.301509817			5

Fig. 5 CNN1			Bonferroni: compare selected pairs		
Young	0.157194203	0.040925653		P<0.001 vs Sene-Control	3
Sene-Control	1	0.144569886			5
Sene-Pue25	0.711613421	0.333769067			5
Sene-Pue50	0.605286672	0.243165877		P=0.023 vs Sene-Control	5
Fig. 5 PDPN			Bonferroni: compare selected pairs		
Young	7.829371859	0.586047405		P<0.001 vs Sene-Control	3
Sene-Control	1	0.057565366			5
Sene-Pue25	1.190467506	0.498024589		P=0.001 vs Sene-Control	5
Sene-Pue50	1.605891794	0.248659236		P=0.012 vs Sene-Control	5
Fig. 6B			Bonferroni: compare selected pairs		
Young	1.12	0.036503424			6
Sene-Control	1.6798	0.156587675		P=0.003 vs Young	6
Sene-Pue50	1.455	0.099002525		P<0.001 vs Sene-Control	6
Fig. 7B			Student t-test		
Control	16.02618045	8.234168567			133
Pue50	31.2224129	10.95156955		P<0.001	155
Fig. 8A			Bonferroni: compare selected pairs		
Control	1	0.075670889			4
+pue50	0.842511618	0.01943555		P=0.012 vs Control	4
+Fulv	0.990097323	0.182162572			4
Fig. 8B			Bonferroni: compare selected pairs		
Control	1	0.272680831			4
+pue50	0.513061226	0.0518114		P=0.022 vs Control	4
+Fulv	0.911758098	0.16062021		P=0.006 vs +Pue50	4
Fig. 8D			Bonferroni: compare selected pairs		
Control	14.14384542	3.608059177			12
+pue50	7.667891614	3.340423255		P<0.022 vs Control	12
+Fulv	12.95855902	3.655076334		P=0.006 vs +Pue50	12

Fig. 8E			Bonferroni: compare selected pairs		
Control	1.820454545	0.197255349			12
+pue50	1.367454545	0.453547652		P=0.006 vs Control	12
+Fulv	1.754	0.247866093		P<0.022 vs +Pue50	12
